# Genetics of dementia: insights from Latin America

**DOI:** 10.1590/1980-57642020dn14-030004

**Published:** 2020

**Authors:** Claudia Ramos, David Aguillon, Christian Cordano, Francisco Lopera

**Affiliations:** 1 Neurosciences Group of Antioquia, School of Medicine, Universidad de Antioquia - Medellín, Colombia.; 2 UCSF Weill Institute for Neurosciences, Department of Neurology, University of California, San Francisco - San Francisco, CA, United States.

**Keywords:** Alzheimer’s disease, frontotemporal dementia, Latin America, genetics, doença de Alzheimer, demência frontotemporal, América Latina, genética

## Abstract

Alzheimer’s disease (AD) and frontotemporal dementia (FTD) are neurodegenerative disorders that result in a significant burden to both patients and caregivers. By 2050, the number of people with dementia in Latin America will increase 4-fold. A deep understanding of the relevant genetic factors of AD and FTD is fundamental to tackle this reality through prevention. A review of different genetic variants that cause AD or FTD in Latin America was conducted. We searched Medline and PubMed databases using the keywords “Alzheimer’s disease,” “frontotemporal dementia,” “mutation,” “America,” and “Latin America,” besides specific Latin American countries. Forty-five items were chosen and analyzed. *PSEN1* mutations are the commonest cause of genetic early-onset Alzheimer’s disease (EOAD), followed by *PSEN2* and *APP* mutations. Genetic FTD can be mainly explained by *GRN* and *MAPT* mutations, as well as *C9orf72 G4C2* repeat expansion. *APOE* ε4 can modify the prevalence and incidence of late-onset Alzheimer’s disease (LOAD), in addition to the cognitive performance in affected carriers.

## RESUMO.

A doença de Alzheimer (DA) e a demência frontotemporal (DFT) são distúrbios neurodegenerativos que causam uma sobrecarga significativa para pacientes e cuidadores. Em 2050, o número de pessoas com demência na América Latina aumentará 4 vezes. Uma compreensão profunda dos fatores genéticos relevantes da DA e da DFT é fundamental para enfrentar essa realidade por meio da prevenção. Foi realizada uma revisão de diferentes variantes genéticas que causam a DA ou a DFT na América Latina. Pesquisamos os bancos de dados Medline e PubMed usando as palavras-chave “doença de Alzheimer”, “demência frontotemporal”, “mutação”, “América” e “América Latina”, além de países latino-americanos específicos. Quarenta e cinco itens foram escolhidos e analisados. As mutações do *PSEN1* são a causa mais comum da doença de Alzheimer genética de início precoce (DAIP), seguida pelas mutações do *PSEN2* e da *APP*. A DFT genética pode ser explicada principalmente por mutações no *GRN*, *MAPT* e expansões repetidas da *C9orf72 G4C2*. O *APOE ε4* pode modificar a prevalência e a incidência da doença de Alzheimer de início tardio (DAIT), mas também o desempenho cognitivo em portadores afetados.

Worldwide, there is nearly one new case of dementia every 3 seconds.^1^ In 2018, the global prevalence of dementia was 50 million cases, and this rate is projected to reach 152 million by 2050.^1^ By 2020, lower-middle-income countries (LMIC) will have 89.28 million people with dementia. In addition, upper-middle-income countries (UMIC) will experience the greatest dementia impact.^2^ In Latin America, dementia is an important public health issue, due to the predicted four-fold increase in subjects with dementia from 2015 to 2050.^2^

Prevention is considered a key factor for tackling this reality, while the study of genetics allows both an accurate characterization of subgroups of patients and personalized prevention.^3^ The purpose of our work is to describe Alzheimer’s disease (AD) and frontotemporal dementia (FTD) genetics in Latin America.

## GENETIC VARIANTS RELATED TO ALZHEIMER’S DISEASE

Presenilin 1 (*PSEN1*) and Presenilin 2 (*PSEN2*) genes carry the active site of the γ-secretase complex.^4^ Besides them, other proteins are encoded by different risk genes related to late-onset AD (LOAD) and involved in Aβ clearance. *SORL1*, *PICALM*, and *CD2AP* genes regulate amyloid precursor protein (APP) endocytosis and the production of A( in the endosomal-lysosomal system.^4^ ^,^ ^5^ On the other hand, *APOE*, *CLU*, *TREM2*, *ABCA7*, *PICALM*, *CD33*, *CD2AP*, and *CR1* are involved in A( elimination.^4^ ^,^ ^5^

In a recent genetic meta-analysis involving 94,437 individuals with LOAD, 20 previous LOAD loci were confirmed.^5^ The authors also described five new genome-wide loci (*IQCK*, *ACE*, *ADAM10*, *ADAMTS1*, *WWOX*), identified when investigating relatives of people diagnosed with AD or dementia. The possible pathways related to these variants involve protein-lipid complex, A( formation and degradation, cholesterol metabolism, tau processing, and immunity. Remarkably, the authors identified a genetic correlation between LOAD, family history of dementia, and education. For example, they found that common genetic LOAD variants were positively correlated to a maternal history of dementia.^5^ Also, they detected a significant negative correlation between AD and educational level: more years of schooling and better cognitive scores behaved as protective factors against LOAD.^5^

## FRONTOTEMPORAL DEMENTIA

FTD is the second most common cause of dementia in individuals under the age of 65. FTD is a neurodegenerative disorder characterized by:

- behavioral difficulties (disinhibition, compulsions, stereotypic movements, hyperorality) and personality and affective changes (loss of empathy, apathy, inertia);
- language and executive dysfunction;
- frontal and/or anterior temporal brain atrophy;
- heritability: 40% of affected patients have a relative with this condition, and the inheritance pattern in 10% of them is autosomal dominant;^6^
- Three genes are often associated with FTD:
- *MAPT*, which is found on chromosome 17q21.31 and encodes the microtubule-associated protein tau protein, *MAPT*;
- *GRN*, which is found on chromosome 17q21.31 and encodes the granulin protein, *GRN*;
- *C9orf72*, which is found on chromosome 9p21.2 and consists of a segment of deoxyribonucleic acid (DNA) with six DNA nucleotides - four guanines and two cytosines (written as GGGGCC).^6^

*MAPT* is an important protein for microtubule stabilization and assembly.^6^ Mutations in this protein cause diseases by 1) altering tau splicing, which affects the normal 3R/4R tau ratio (usually the 3R/4R ratio in the adult human brain is very stable); 2) promoting cytoplasmic tau aggregation; and 3) causing tau hyperphosphorylation, which generates microtubule instability.^6^ ^,^ ^7^ *MAPT* mutations are related to several phenotypes, mainly the behavioral variant FTD (bvFTD).^6^ Notoriously, *MAPT* mutation carriers can develop episodic memory impairment.^6^ Brain neuroimaging can show symmetric involvement of anterior temporal, lateral prefrontal, and orbitofrontal regions.^6^ The neuropathology of *MAPT-*mutation syndromes is characterized by frontotemporal lobar degeneration (FTLD), a neurodegenerative process involving neuronal loss and gliosis of the frontal and temporal brain regions, in the presence of hyperphosphorylated tau inclusions.^8^ Alternative splicing of *MAPT* generates six different tau isoforms; the inclusion of exon 10 produces tau isoforms with either four (4R tau) or three (3R tau) microtubule-binding domain repeats. In FTLD-tau, Pick’s disease is characterized by predominant deposition of 3R tau aggregates, whereas corticobasal degeneration (CBD) and progressive supranuclear palsy (PSP) are 4R tauopathies.^8^

*GRN* is a protein necessary to activate signaling cascades for neuronal growth, inflammation, and wound repair.^6^ ^,^ ^8^ Mutation carriers have a 50% decrease in *GRN* mRNA progranulin levels in plasma and cerebral spinal fluid (CSF).^8^ *GRN* mutations are usually related to bvFTD.^6^ As in some *MAPT* cases, *GRN* carriers can present episodic memory impairment, besides neuropsychiatric symptoms, such as psychosis and isolation.^6^ Neuroimaging shows asymmetric atrophy of frontotemporal regions.^6^ ^,^ ^8^ Carriers’ pathology is characterized by a pathologic form of TAR DNA-binding protein 43 (TDP-43), distributed in cytoplasmic aggregates leading to microtubule phosphorylation, ubiquitination, and degradation.^8^ Specifically, the neuropathology of *GRN* mutation carriers is the TDP-43 type A.^8^

As mentioned before, the *C9Orf72* gene has a hexanucleotide repeat expansion in its first intron: GGGGCC. Normal alleles have three to thirty repeat units, while people with the repeat expansion mutation can present hundreds to thousands of them.^9^ Its encoded protein, C9orf72, has been involved in coupling cytoskeleton modulation and autophagy with endocytosis.^9^ *C9Orf72* mutations have been related to bvFTD, frontotemporal dementia-amyotrophic lateral sclerosis (FTD-ALS), nonfluent variant primary progressive aphasia (nfvPPA), corticobasal syndrome (CBS), and rarely semantic variant primary progressive aphasia (svPPA).^6^ As with *GRN*, *C9Orf72* carriers can develop neuropsychiatric symptoms, such as psychosis and anxiety. Moreover, as in *MAPT* and *GRN*, these patients can present visual and verbal episodic memory impairment.^6^ Neuroimaging shows symmetric and sometimes minimal atrophy of the cerebral hemispheres, thalamus, and cerebellum.^6^ TDP-43 type A can be found in *C9Orf72* carriers, but the commonest pathology in this population is TDP-43 type B, consisting of less neuronal cytoplasmic inclusions and dystrophic neurites in both cortical layers and lower motor neurons. Another interesting pathological finding is the presence of ubiquitin-positive, TDP-43-negative inclusions in the cerebellum, neocortex, and hippocampus.^8^

Other important mutations associated with FTD include *VCP*, *CHMP2B*, *TARDBP*, *FUS*, *SQSTM1*, *UBQLN2*, *TDK1*, *TREM2*, *CHCHD10*, and *PRNP*. Some of them will be mentioned in the next sections.

## METHODS

We searched the PubMed and Medline databases using the keywords “Alzheimer’s disease,” “frontotemporal dementia,” “mutation,” “America,” and “Latin America,” filtering for “human” research, without restricting dates for relevant articles. The initial search produced 205 hits. We searched specific Latin American countries to ensure the articles found were relevant to specific regions. After reading the abstracts and discarding articles unrelated to the genetics of AD and FTD in Latin America and the Caribbean, we selected and reviewed a total of 45 studies.

## ARGENTINA

### Alzheimer’s disease

#### Presenilin 1 mutations

#### T119I

Two members of an Argentine family of Italian descent underwent both clinical evaluation and genetic testing, and a mutation in *PSEN1 T119I* was found.^10^

The proband’s first cognitive complaints happened when he was 49 y.o. Next, the patient developed depression after a sibling’s death. Finally, the participant was diagnosed with Alzheimer’s dementia nine years after the memory complaints (58 y.o.). One of the proband’s parents and several uncles and cousins were also affected by the same condition. After DNA analysis, Itzcovich et al. found a heterozygous C>T transition at position c.356 of *PSEN1*, a variant that predicts a threonine-to-isoleucine substitution at codon 119 (p.T119I), located in the first extracellular loop of the protein (HL-I loop).

Clinical symptoms include memory, executive, and attention impairment.^10^ Neuroimaging showed bilateral amyloid accumulation in the neocortex (frontal, parietal, and lateral temporal lobes), cingulate, and striatum.^10^

Mean age of onset was 56 years, but one family member was diagnosed with LOAD at the age of 71 years, suggesting that this mutation can cause autosomal dominant AD of both early and late onset in this population.^10^

#### M146L

This autosomal dominant mutation was found in an Argentine family and described in 1998. It consists of an A>T transversion in codon 146 of *PSEN1*, a variant that causes a methionine-to-leucine substitution.^11^ Mean age of onset was 38.9±3.9 years, and mean age at death was 41.7 through 51 years. Carriers developed symptoms such as memory loss, early language impairment, myoclonus, and cerebellar signs, besides some neuropsychiatric symptoms, including aggressiveness and apathy. All subjects in that report were *APO(3* homozygotes.

#### M146V

Riudavets et al.^12^ described an Argentine family originally from Portugal. This case of familial dementia associated with a PS-1 M146V mutation presented with typical clinical features of FTD (gradual apathy, disinhibition, executive dysfunction, anomia, and memory loss followed by extrapyramidal manifestations, including rigidity, akinesia, and movement disorders, without tremor). Afterward, the proband clinical state worsened and progressed to myoclonus, seizures, and mutism.

#### Presenilin 2 mutations

#### N141I

Researchers from the Centro de Neuropsiquitría y Neurología de la Conducta (CENECON), Universidad de Buenos Aires, characterized these clinical phenotypes in two Argentine pedigrees with clinical symptoms of early-onset AD (EOAD) and found *PSEN2 Asn141Ile* (*N141I*) in all affected subjects.^13^ The carriers developed early episodic memory impairment, visuospatial impairment, limited verbal fluency, and executive dysfunction, as well as neuropsychiatric symptoms like apathy. Noteworthily, epilepsy was rare.^13^

Even though *N141I* is the most frequent *PSEN2* mutation, this research was the first *PSEN2 N141I* report in South America. Moreover, these families share the same Volga German (VG) ancestry, that is, their ancestors moved from Germany to the southern Volga region in the 1760s, generating the founder effect of this mutation.

Affected members’ mean age of onset was 52.7±3.2 years, the mean age at death was 60.7±3.6 years, and the mean disease duration was 7.9±3.1 years. This mutation has a penetrance of 100%.

The *APOE* polymorphism phenotype of the carriers was: (3/(3 and (2/(3, with no impact on onset and progression of the disease, and (4/(4, although determining the impact of this last genotype at the age of onset or disease progression was not possible.

### Frontotemporal dementia

#### Allele repeat expansions

#### C9ORF72 G4C2

The first case of FTD due to *C9orf72 G4C2* repeat expansion in Argentina was described in 2016.^14^ It involved a 51-year-old proband who developed **behavioral disorders** (anxiety, aggressiveness due to feeling offended in public places, psychotic ideation, and delusions), followed by language difficulties and cognitive impairment for over 3 years. The psychotic symptoms, in addition to a family history of **parkinsonism** and **ALS**, made the clinicians suspect *G4C2* hexanucleotide repeat expansion in *C9ORF72*. The presence of an expanded *G4C2* allele in the patient was then confirmed (more than 50 repetitions).

Itzcovich et al. genotyped the *C9orf72 G4C2* repeat in patients with FTD (n=33), ALS (n=50), and age-matched healthy controls (n=73) in Argentina.^15^ They found a male to female ratio of 1:1 in the ALS group, while 60.6% of FTD individuals with *C9orf72* repeat expansion were women. The overall G4C2 expansion frequency among FTD cases was 18.2% (6 out of 33 FTD cases). Among ALS patients, only two cases (one with positive family history and one sporadic) carried a G4C2 expansion. In the six G4C2 expansion carriers with FTD, five had bvFTD and just one had primary progressive aphasia; curiously, most of these patients (3 out of 5 or 60% of this group) showed motor neuron disease during follow-up.

#### MAPT mutations

#### P301L

In 2017, Gatto et al. reported an intrafamilial variable phenotype in a family with a missense mutation (p.P301L; rs63751273) in exon 10 of the *MAPT* gene.^16^ This family of Basque ancestry had 26 members over 6 generations and 9 affected individuals. The two living subjects analyzed had different phenotypes: CBS (1 female) and FTD (1 male), while the 7 deceased family members over 4 generations, whose cases were described as early-onset dementia, presented with apathy and disinhibition as the main symptoms. The CBS proband also had cognitive complaints that were evident in the neuropsychological tests, with attention deficit and dysexecutive impairment.

During the follow-up, the CBS proband developed gait disturbance, high risk of fall, worsening of motor symptoms in her right arm (focal dystonia and alien limb syndrome), myoclonus, hyperreflexia, apraxia, and frontal release signs.^16^ Attention and executive function worsened, and she developed problems with memory and language.^16^

The second case (FTD) involved a man with symptoms such as logorrhea, tangentiality, disinhibition, inappropriate behavior such as waking his son up at night just to talk (the authors of the report considered it a lack of empathy), isolation, compulsions (alcoholism), repetitive behaviors (eating the same meals every day), emotional apathy (he seemed not to care about his difficult financial situation), and grooming neglect.^16^ Neuropsychological tests led to the conclusion that he had attention problems and executive impairment (as in the proband’s case). Finally, a brain magnetic resonance imaging (MRI) showed asymmetrical frontal atrophy of the frontal lobes, a finding highly suggestive of bvFTD.^16^

## BRAZIL

### Alzheimer’s disease

#### Presenilin 1 variants

Barbosa Abdala et al.^17^ performed a mutational screening of *PSEN1* among 53 samples of people with a family history of AD from Rio de Janeiro. Four variants were identified: two missense variants (*rs63750592*; *rs17125721*) and two intronic variants (*rs3025786*; *rs165932*). Prediction results and other articles showed that *rs17125721* (*Glu318Gly* or *E318G*) is a risk variant and not a monogenic cause of AD, while *rs63750592* (R35Q) is a variant of unclear pathogenicity.

A subsequent case-control study evaluated the role of *rs17125721* or *E318G* on AD risk. A sample of 120 sporadic AD cases and 149 healthy older adult controls was analyzed, and a risk association for *s17125721* or *E318G* was identified in familial AD cases (*Odds Ratio* - OR=6.0; 95% confidence interval [95%CI] 1.06-33.79; p=0.042); no statistical association was found between the *APOE* ε4 allele and *rs17125721* or *E318G*.^17^

#### APOE4

In 1997, Almeida and Shimokomaki^18^ examined the association between the *APOE* ε4 allele and AD, which was being reported in developed countries. After analyzing a sample of 55 AD patients and 55 controls, they found that *APOE4* was more frequent among the AD patients (20.9% versus 8.9%; p=0.038), establishing that one *APOE* ε4 allele was enough to increase by 2.63 times the odds of being diagnosed with dementia. Interestingly, they also noticed a trend toward earlier onset among *APOE* ε4 carriers.

Recently, De Luca et al.^19^ explored the influence of *APOE* ε4 on the age of onset of AD. To that end, they analyzed three groups from three different sources: 414 patients from Italy, 135 patients from Brazil, and 376 patients from the Alzheimer’s Disease Neuroimaging Initiative (ADNI) consortium. *APOE* ε4 showed a significant anticipatory effect among people with LOAD in the three samples. The Brazilian group presented a particularly significant *APOE* ε4 anticipatory effect (p=0.001; beta= -10.2; 95%CI -11.0 to -9.4).

Besides *APOE* ε4, the authors found other polymorphisms related to LOAD, such as *SOR1*, *GAB2*, and *GSK3B*. Since none of these last variants are considered etiological factors, despite being able to interact with other genetic forms to modify the risk of AD, Izzo et al.^20^ conducted a study to explore the interaction of these polymorphisms with *APOE* ε4. The authors found an association between the *SORL1* GG genotype and AD (OR=2.07; 95%CI 1.17-3.68; p=0.047), regardless of the presence of *APOE* ε4. They also discovered a positive association between the *GAB2* GG genotype and AD (OR=1.8; 95%CI 1.01-3.18; p=0.021), which was higher in *APOE* ε4 carriers (OR=5.08; 95%CI 1.45-18.98; p=0.006). Finally, the authors identified an association between the *GSK3B* GG genotype and AD (OR=2.48; 95%CI 1.19-5.20; p=0.018), which was higher in the absence of the ε4 allele. Also, they found a protective effect related to the A allele of *GSK3B*, irrespective of the *APOE* status.

### Frontotemporal dementia

#### Allele repeat expansions

#### C9ORF72 G4C2

In 2012, this expansion was described in relatives with several affected members.^21^ Similar to previous reports, the main clinical syndromes seen in this *C9orf72* family were bvFTD and ALS. In many members, subtle personality changes started decades before dementia was diagnosed. Interestingly, one of the subjects was diagnosed with bvFTD at an earlier age than the previous generation, raising the hypothesis of an anticipation phenomenon. Two patients had inflammatory bowel disease. As in other *C9orf72* cases, hallucinations were a characteristic symptom (rare in sporadic bvFTD). Furthermore, neuroimaging showed a moderate posterior pattern of atrophy, described as more important parietal and occipital atrophy with reduced temporal tissue loss when compared to sporadic bvFTD.

In 2016, Chadi G et al.^22^ evaluated the clinical features of subjects carrying *C9orf72* repeat expansions within a Brazilian cohort of ALS patients and identified a patient diagnosed with FTD.

In 2018, a study about the frequency of this expansion in the Brazilian population was published.^23^ Among the 471 patients analyzed (404 with ALS/motor neuron disease, 67 with FTD, and 63 healthy controls), the highest frequency of the expansion was in the FTD-ALS group (50% of familial and 17.6% of sporadic cases), followed by 5% of pure ALS/motor neuron disease patients (11.8% of familial and 3.6% of sporadic cases) and 7.1% of pure familial FTD individuals.

#### GRN, MAPT, and TARDBP mutations

In 2016, Takada et al. investigated the frequencies of *GRN* and *MAPT* mutations in FTD cohorts from two Brazilian dementia research centers.^24^ They analyzed blood samples from 76 probands diagnosed with bvFTD (n=55), svPPA (n=11), or nfvPPA (n=10). A total of 25% of the cohort had at least one relative affected by FTD.

The authors found *GRN* mutations in seven probands (9.6%), and *MAPT* mutations in two probands (7.1%). *GRN* and *MAPT* mutations explained 31.5% and 10.5% of the familial cases, respectively. In individuals with *GRN* mutations, three novel *GRN* mutations were identified (patients with bvFTD): ***p.Q130X*** ,p.D317Afs*12 , and p.K259Afs*23 . Other *GRN* mutations found in that sample were: p.Q257Pfs*27 (proband with nfvPPA), *p.Q300X* (proband with nfvPPA), p.V200Gfs*18 (proband with nfvPPA/mixed PPA), and p.S301Cfs*61 (proband with bvFTD).

Subjects with the *Q130X GRN* mutation (or some of their relatives) showed memory difficulties, especially of the semantic type, language impairment (nfvPPA), irritability, apathy, and other bvFTD symptoms, such as diet changes, loss of empathy, and executive impairment.^24^ One patient developed delusions. On the other hand, a *GRN D317Afs* mutation carrier showed bvFTD symptoms that included disinhibition, impulsive behavior, and apathy at the age of 63. Later, the patient developed motor impairment (parkinsonism). Asymmetric but bilateral atrophy was identified in the brain MRI, and pathology revealed the involvement of frontal, parietal, and temporal lobes. His mother also had bvFTD and motor symptoms.^24^ Finally, a person with *K259Afs GRN* mutation and some relatives developed anomia, logorrhea, apathy, disinhibition, diet changes, and asymmetric brain atrophy, especially of the temporal lobes.^24^

Regarding *MAPT*, an *Asn279Lys* (*N279K*) mutation was found in a 45 y.o. proband with bvFTD and PSP. The other proband had a g.120998 cytosine to thymine change, an intronic variant that affects the alternative splicing of exon 10 (IVS10+16 C>T), which does not lead to a protein change but could explain the bvFTD phenotype of this subject.^24^

*TARDBP* is a gene in chromosome 1 that encodes a protein called TDP-43, an important riboprotein with functions such as mRNA stabilization, transcription regulation, and alternative splicing.^6^ ^,^ ^25^ Mutations in this gene have been associated with FTD, FTD-ALS, and ALS.^25^ In 2012, Machado-Costa screened a sample of 47 FTD cases for *TARDBP* mutations and found a *p.I383V* mutation in a proband diagnosed with svPPA at the age of 54.^25^ The mutation carrier also had neuropsychiatric symptoms like irritability, apathy, disinhibition, and obsessive-compulsive behavior; his brain MRI showed bilateral temporal atrophy.

#### Valosin-containing protein mutations

Katsuyuki-Shinjo et al.^26^ reported a Brazilian family with inclusion body myopathy with early-onset Paget disease and frontotemporal dementia (IBMPFD), an autosomal dominant disease linked to chromosome 9p21-p12. This condition is attributed to missense mutations in the valosin-containing protein (***VCP*** ) gene, whose protein has been involved in proteolysis.

Ten family members from three generations were evaluated. The proband’s (male, 58 y.o.) first symptom was distal progressive muscle weakness of the four limbs. Myopathy was confirmed by electromyography and muscle biopsy. Afterward, he developed personality changes and cognitive impairment. MRI and single-photon emission computed tomography (SPECT) showed atrophy and hypoperfusion in the frontal and temporal brain areas, specifically. A computed tomography (CT) revealed increased density, coarse trabeculation, and cortical thickening of the cervical spine. His mother had AD, and his father had chronic asymmetric limb muscle weakness. He tested positive for *VCP* mutation: *c.290G>A*, *p.Gly97Glu*, or *p.G97E*. Two of his siblings and a nephew had this mutation as well.^26^

Abrahao et al.^27^ also described three clinical cases of relatives with intrafamilial phenotype variability: each participant had either myopathy with rimmed vacuoles, ALS, or FTD, but none had Paget disease of bone (PDB). After the whole-exome sequencing, they confirmed the segregation of a novel mutation, *p.Asn91Tyr* or *N91Y*, in an autosomal dominant pattern. The proband with FTD was diagnosed with probable bvFTD, with onset at the age of 66.

Fanganiello et al.^28^ also described a family with IBMPFD. The proband presented mild progressive proximal myopathy, PDB (onset at the age of 55 years), behavioral disturbances, and cognitive impairment (onset at the age of 56 years). The patient developed FTD with features of both bvFTD and semantic dementia. Mutation analysis of the propositus revealed a heterozygous nucleotide transition in exon 3 (c.277C → T) of the *VCP* gene, resulting in an arginine substitution with cysteine in codon 93.

#### Prion protein mutations

*PRNP* is a gene encoded by chromosome 20, responsible for the prion protein (PrP), a 208 amino acid membrane glycoprotein, whose exact function is unclear, but that could participate in processes such as neuronal protection, cellular adhesion, cell signaling, and circadian rhythm control.^29^

In 1996, Nitrini et al.^30^ reported a *PRNP* mutation, *Thr183Ala* or *T183A*, which had an autosomal dominant genetic pattern and led to a rapidly progressive FTD (disease duration: 4.2±2.4 years) at the age of 44.8±3.8 years. The proband showed apathy, memory problems, time and visuospatial disorientation, slurred speech, parkinsonism, and frontal release signs. The electroencephalogram was normal, but he had diffuse cortical atrophy. The brain pathology revealed spongiform changes and neuronal loss in the putamen, besides minimal gliosis in the remaining affected regions. Other family members were also affected by this condition.

## CHILE

### Alzheimer’s disease

#### M146I

In 2010, Sinning et al.^31^ reported an extended Chilean pedigree affected by EOAD for 4 generations. The cause in all affected members was a heterozygous G to C transversion at position 438 of the mRNA in *PSEN1* (*14q24*), which results in a methionine to isoleucine substitution at position 146 of the protein (*M146I*). The age of onset of dementia ranged between 38 and 42 years. They had early neuropsychiatric manifestations, such as anxiety and depression, and other neurological issues, including myoclonus and generalized epilepsy.

#### Frontotemporal dementia

#### C9Orf72

In 2017, Miranda et al.^32^ described the case of a 77 y.o. woman who developed symptoms such as apathy, less fluent language, anomia for common items, and circumstantial speech when she was 68 y.o. Later, she developed inattention, problems with semantic memory and frontal test, and nfvPPA, besides parkinsonism without rest tremor or rigidity. Other relatives also had a history of dementia, parkinsonism, and ALS (several brothers and a sister, the mother, and many maternal aunts and uncles). Severe atrophy of frontal and temporal lobes was identified in this proband. Genetic testing showed a GGGGCC *C9orf72* abnormal expansion (approximately 60 repeats). This was the first described case of familial FTD due to *4G2C C9Orf72* repeat expansion in Chile.

## COLOMBIA

### Presenilin 1 E280A

*E280A* is a glutamic acid to alanine mutation at codon 280 of the *PSEN1* gene. The consequence is an early-onset familial AD at a mean age of 49 years, with **fully penetrant autosomal dominant transmission**.^33^ It was first discovered in a family from the state of Antioquia, Colombia, with over 6000 members subsequently identified and described.

*PSEN1 E280A* carriers will undergo four AD stages:

- asymptomatic pre-mild cognitive impairment (MCI) (median age: 35 y.o.; 95%CI 30-36);
- symptomatic pre-MCI (38 y.o.; 95%CI 37-40);
- MCI (44 y.o.; 95%CI 43-45);
- dementia (49 y.o.; 95%CI 49-50).^34^

In affected carriers, the main neurological features are: memory impairment (100%), behavioral changes (94%), language impairment (such as aphasia, 81%), headache (migraine and non-migraine, 73%), gait difficulties (65%), seizures and myoclonus (45%), cerebellar signs (19%), and parkinsonism (19%).^35^ This symptomatology is supported by pathological changes, such as brain atrophy, cerebellar damage, and severe amyloid and tau-related pathology.^35^

Regarding *APOE* polymorphisms in this population, Pastor et al.^36^ found no association between *APOE* ε4 and age at disease onset in *PSEN1 E280A* mutation carriers. On the other hand, *APOE* ε2 delays the age of onset, making it a protective factor.^37^

### Presenilin 1 I416T

*I416T* is the cause of dementia in the second largest family with EOAD in Antioquia, Colombia. Ramírez-Aguilar, Acosta-Uribe et al.^38^ recently found the variant *c.1247T.C* in codon 416 of *PSEN1*, which causes isoleucine to threonine substitution and impairs a highly conserved residue in the 8^th^ transmembrane domain of presenilin 1.

The age of onset was 42.35 years (standard deviation [SD] 6.28) for memory complaints, 47.6 years (SD 5.83) for MCI, and 51.6 years (SD 5.03) for dementia. Among the several neuropsychiatric symptoms are depression, anxiety, delusions, hallucinations, and insomnia. Besides memory impairment, the affected carriers develop myoclonus and tonic-clonic seizures. Neuropsychological symptoms include amnestic MCI, and, interestingly, these patients show better performance in language and attention than praxis and executive function. *APOE* ε4 does not behave as an age modifier in this population.

### APOE3ch

In 2019, Arboleda-Velasquez et al.^39^ described a mutation in *APOE3* called *APOE3ch*, caused by an arginine to serine substitution at amino acid 136 (*R136S*). The mutation was found in homozygous form in a *PSEN1 E280A* carrier who developed MCI in her seventies, even though the median age of MCI onset is usually 44 y.o.^40^ The subject had problems with recent memory, and her neurological exam was normal. Neuroimaging techniques such as positron emission tomography (PET) showed an important amyloid-β burden, with a distribution volume ratio (DVR) of 1.96 - much higher than that of younger people with MCI and the same *PSEN1* mutation (DVRs 1.49-1.60). Nevertheless, PET tau burden and neurodegeneration were limited (only medial temporal and occipital regions were affected), and the fluorodeoxyglucose PET did not detect the glucose metabolism abnormalities typical of people with this familial AD type. Also, her MRI showed atrophy similar to that of carriers with MCI in their forties.

The *APOE3ch* mutation is located in a region that plays a role in binding to lipoprotein receptors and heparan sulfate proteoglycans (HSPGs).^41^ HSPGs seem to promote amyloid-β aggregation and neuronal tau uptake through processes like APOE binding.^42^ APOE3ch presents the lowest heparin-binding ability (compared to other *APOEs*), becoming a protective genetic variant against the early onset of autosomal dominant AD when a person has homozygosity.^39^

The authors concluded that *APOE3ch* homozygous people might be resistant to the clinical onset of AD by limiting tau pathology and neurodegeneration despite the significant amyloid-β burden in those subjects.^39^

### Other mutations

In 2001, Arango et al.^43^ published a systematic genetic analysis of *APP*, *PSEN1,* and *PSEN2* mutations in a Colombian sample of 76 subjects with AD, identifying several mutations. In *APP,* they found a silent *Gly708 (G708*) mutation in a proband with sporadic AD. In *PSEN1*, they discovered a new *PSEN1* mutation, *Val94Met*, in a subject with sporadic AD, which was absent in the 53 asymptomatic controls; they also identified other three *PSEN1* mutations: *Ile143Thr* (*I143T*, autosomal dominant), *Glu280Ala* (*E280A*, autosomal dominant and previously described), and *Glu318Gly* (*E180G*, in sporadic and familial cases). Nowadays, *PSEN1 E318G* is considered a risk modifier of this disease.^44^ Finally, in *PSEN2,* they found two silent mutations: *Pro129* or *P129* (in a patient with autosomal dominant AD) and *Ser236* or *S236* (in three sporadic cases). *APOE* polymorphisms were not measured, which was considered a limitation of the study.

In the state of Valle del Cauca, in western Colombia, a *PSEN1 Pro117Ala (P117A)* mutation was found in eight patients. This mutation causes autosomal dominant EOAD during the fourth decade of life (Table 1).^45^ The first *PSEN1 Pro117Ala* mutation case reported involved three patients from the same family in France with early progressive ataxia (which occurs 1-7 years after the onset of cognitive decline) and dementia.^46^ Ataxia was not reported in the Colombian pedigree with this mutation.

**Table 1.** Colombian mutations (besides Presenilin 1 *E280A*, Presenilin 1 *I416T*, and *APOE3ch*).

Mutation	State or department	Phenotype	Age of onset (years)
*APP Gly708*	Cundinamarca	Silent mutation, but the proband had sporadic Alzheimer’s disease	71
*PSEN1 Val94Met*	Cundinamarca	Sporadic Alzheimer’s disease	53
*PSEN1 Ile143Thr*	Cundinamarca	Autosomal dominant Alzheimer’s disease	30
*PSEN 1 Glu318Gly*	Cundinamarca	Sporadic and familial cases (the mutation is a risk modifier)	65.8 (49-86)
*PSEN1 Pro117Ala*	Valle del Cauca	Autosomal dominant Alzheimer’s disease	Fourth decade of life
*PSEN2 Pro129*	Cundinamarca	Silent mutation, but the proband had autosomal dominant Alzheimer’s disease	62
*PSEN2 Ser236*	Cundinamarca	Silent mutation, but the probands had sporadic Alzheimer’s disease	83.2 (78-88)
*TREM2 Trp198X*	Antioquia	PLOSL and early-onset dementia (autosomal recessive)	FTD-like syndrome, at 47

PSEN1: Presenilin 1; PSEN2: Presenilin 2; APP: amyloid precursor protein; PLOSL: polycystic lipomembranous osteodysplasia with sclerosing leukoencephalopathy; TREM2: triggering receptor expressed on myeloid cells 2; FTD: frontotemporal dementia.

Triggering receptor expressed on myeloid cells 2 (*TREM2*) is an important protein for the phagocytosis of apoptotic neuronal cells by microglia in the brain. In 2013, Giraldo et al.^47^ described a *TREM2 W198X* mutation that results in polycystic lipomembranous osteodysplasia with sclerosing leukoencephalopathy (PLOSL), an autosomal recessive condition related to early-onset dementia (an FTD-like syndrome). The clinical profile of these patients consisted of compulsive tobacco use and alcohol consumption, convulsive disorder, and neuropsychiatric symptoms, such as obsessions, compulsions, apathy, impulsivity, disinhibition, and cognitive impairment (bradyphrenia, executive dysfunction, apraxia, and memory loss). The frontal atrophy identified in the brain MRI was bilateral but asymmetric and worse on the right side. The authors of this report concluded that *TREM2* might be a risk factor for neurodegeneration and suggested that other neurodegenerative disease cohorts should be examined for its genetic variants.

In 2018, Arboleda-Bustos et al.^48^ studied the association between several *TREM2* polymorphisms and LOAD. They found a relationship with the *TREM2* arginine to histidine substitution at amino acid 47 (*R47H*) in a sample of 358 AD cases and 329 controls; specifically, this variant was identified in six LOAD probands: five (5/558 or 1.4%) heterozygous and one (1/358 or 0.28%) homozygous. No control presented this polymorphism. The difference in the *R47H* allele frequency between cases and controls was statistically significant (p=0.010). They also detected a higher frequency of *APOE4* in AD cases (ε3/ε4 genotype presented an OR=1.738; 95%CI 1.233-2.450; p=0.0016, and ε4/ε4 genotype showed an OR=10.568; 95%CI 3.197-34.932; p=0.0001). Out of the five heterozygous *R47H* carriers, three were *APOE* ε3/ε3 and two, *APOE* ε3/ε4; the homozygous subject was *APOE* ε3/ε4, with a family history of LOAD, and age of onset of 66 y.o. The authors concluded that *TREM2 R47H* could be an important LOAD risk factor, but more studies are necessary to corroborate the relationship described.^48^

In Colombia, a recent study about LOAD genetics has also been performed.^49^ It evaluated the association of 14 single-nucleotide polymorphisms in genes that have been connected to LOAD and confirmed this relationship through a genome-wide association study. Indeed, significant associations were identified for variants in *BIN1* (*rs744373*; OR=1.42), *CLU* (*rs11136000*; OR=0.66), *PICALM* (*rs541458*; OR=0.69), *ABCA7* (*rs3764650*; OR=1.7), and *CD33* (*rs3865444*; OR=1.12). Likewise, a significant interaction effect was observed between *CLU* and *CR1* variants and *APOE(*. These results reflect the importance of gene-gene interactions for the etiology of neurodegenerative diseases.

Table 1 presents a summary of the mutations described in Colombia different from *PSEN1 E280A*, *PSEN1 I416T*, and *APOE3ch* ones.

## CUBA

### Alzheimer’s disease

#### Presenilin 1 mutations

#### L174M

This variant was found in a family of 281 members over six generations, with the proband descending from a Spanish founder.^50^ The mutation results from a C-to-A change detected in exon 6 of the *PSEN1* gene. Mean age of onset was 59 years. The patients had dyscalculia, attention difficulties, visuospatial disorientation, emotional apathy, anosognosia, and slowing speech. Nevertheless, the most relevant features were memory impairment and ischemic episodes. The *APOE* genotype varied: ε3/ε3, ε3/ε4, and ε4/ε4. The subjects with ischemic attack also had *APOE* ε4, an allele related to a possible increase in cerebrovascular risk. Brain examination confirmed amyloid pathology.

#### APOE genotype

The effects of ethnic identity, genetic admixture of *APOE* genotypes, and its association with dementia prevalence and incidence have been explored. In a 10/66 study report, Llibre-Rodriguez et al.^51^ described the *APOE* status in 2520 participants, with genetic admixture in 235 dementia cases and 349 controls. They concluded that *APOE (*4 allele carriage is associated with an increased prevalence and incidence of dementia in populations characterized by African/European admixture. The associations of *APOE* ε4 allele carriage with prevalence were stronger than those with incidence. The explanation for this phenomenon could be a possible earlier age of onset for *APOE* ε4 carriers.

## MEXICO

### Alzheimer’s disease

#### Presenilin 1 mutations

#### L171P

In 1998, Ramirez-Dueñas et al.^52^ reported a new mutation in Mexican families with EOAD (36-40 y.o.), explained by a thymine to cytosine mismatch in exon 7 (nucleotide 760 of cDNA), which leads to the *Leu171Pro* mutation. This mutation was considered pathogenic.

#### A431E

Twelve unrelated families with EOAD from Jalisco, Mexico, were analyzed in 2005.^53^ The *Ala431Glu* mutation in exon 12 of *PSEN1* was found in nine (75%) of these families, with an autosomal dominant inheritance. Also, 15 families were identified in Guadalajara (n=2), Chicago (n=1), and Southern California (n=12).^54^

The ages of onset ranged between 34 and 48 years (mean age of 40 years). A phenotypic variability characterized by spastic paraplegia, myoclonus, aphasia, and psychiatric symptoms (mostly depression and personality changes) was observed. Neuroimages (CT and MRI) showed cortical and subcortical atrophy, while pathology confirmed the diagnosis of AD.

#### APOE genotype and cognition

In 2008, Villalpando-Berumen et al.^55^ conducted a cross-sectional analysis of a cohort study to determine the influence of *APOE* ε4 on the cognition of older multiracial Mexican adults. They found no increased risk for AD in *APOE* ε4 carriers, but its presence seems to be associated with worse performance in a long-term visual memory test among subjects with dementia. The authors concluded that *APOE* ε4 modifies the clinical expression of AD.

## PUERTO RICO AND DOMINICAN REPUBLIC

### Presenilin 1 G206A

A family case series and a cohort study were conducted in New York, Dominican Republic, and Puerto Rico.^56^ ^,^ ^57^ The researchers identified a G-to-C nucleotide change that causes a glycine to alanine amino acid substitution at codon 206, exon 7 of *PSEN1*, defining a ***G206A* mutation**. These families have a possible common ancestor.

Besides cognitive impairment, the affected carriers presented depression, vascular dementia, and other FTD-like symptoms, such as psychosis, motor impairment, epilepsy, and ataxia.^57^

Age of onset slightly varied between the Caribbean countries: 54.7 (SD: 7.1) y.o. for Dominicans and 59.6 y.o. (range: 46-67, with an 81 y.o. outlier) for Puerto Ricans. *APOE* ε4 had no effect on the age of onset, while *SNX25*, a gene with a potential role in regulating membrane protein trafficking excess levels of amyloid-β in individuals with the *PSEN1 p.G206A* variant, may be a biologically relevant modifier of the age of onset.

## URUGUAY

### PRNP G114V

In 2005, Rodriguez et al.^58^ described an Uruguayan family with a mutation in the *PRNP* gene, *Gly114Val* or *G114V*, related to bvFTD and motor neuron disease. The affected members had memory loss and neuropsychiatric symptoms, such as panic disorder, aggressiveness, visual hallucinations, grooming neglect, apathy, and stereotypic behavior, besides speech disorder, corticospinal syndrome, cerebellar signs, myoclonus, apraxia, and asomatognosia. This kind of dementia has a very early onset (second and third decade of life) and sometimes progresses rapidly. Electroencephalogram and brain MRI revealed diffuse brain damage. The frontal biopsy in one of the patients showed spongiosis, gliosis, and neuronal loss in the absence of amyloid deposition. Not all carriers developed the disease, which is suggestive of a mutation with incomplete penetrance.

**Figure 1.**
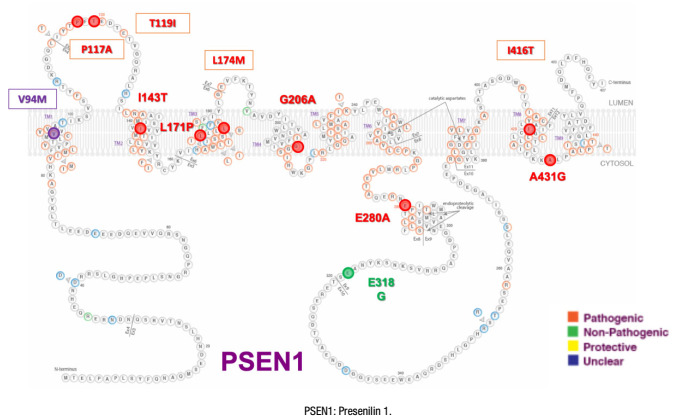
Latin American Presenilin 1 mutations.

**Figure 2.**
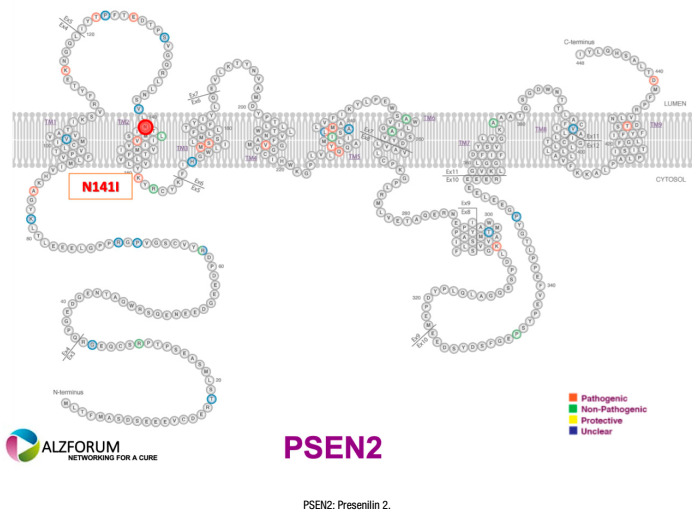
Latin American Presenilin 2 mutations.

**Figure 3.**
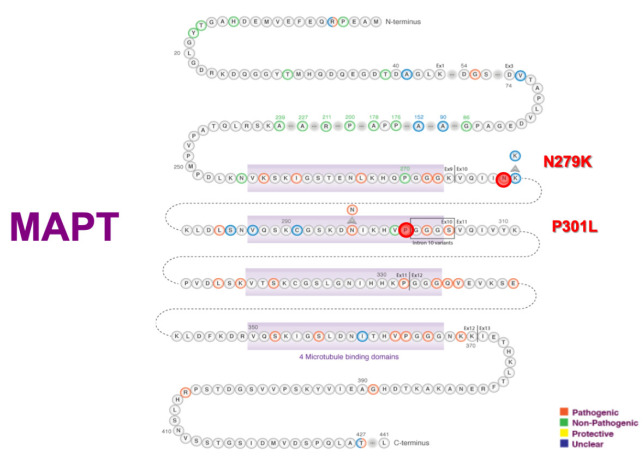
Latin American MAPT mutations.

**Figure 4.**
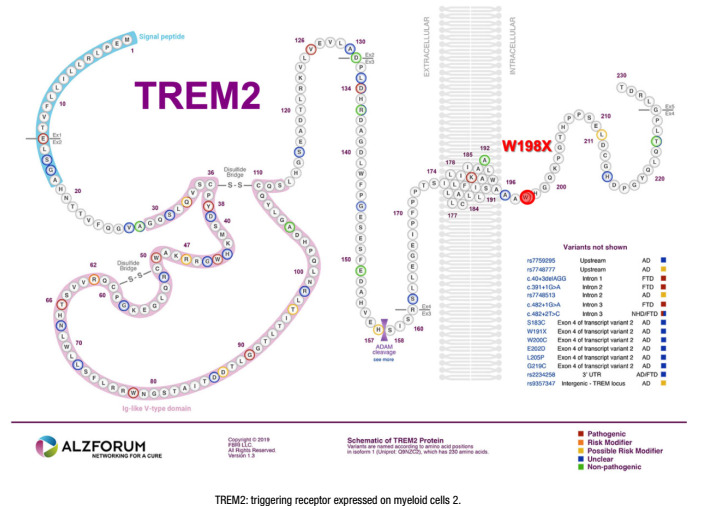
Latin American triggering receptor expressed on myeloid cells 2 mutations.

Figures 1, 2, 3, and 4 present the Latin American mutations in *PSEN1*, *PSEN2*, *MAPT*, and *TREM2*, respectively. As described above, many lines of evidence point to a genetic basis for the development of AD, both in its early-onset forms and in the much more common late-onset form. The finding of different mutations in several genes related to AD and FTD provides a great opportunity for future studies based on primary and secondary prevention. Longitudinal follow-up studies of our own populations are necessary, as well as a continuous search for new cases of families with dementia in Latin America.
